# Changes in weight status, quality of life and behaviours of South Australian primary school children: results from the Obesity Prevention and Lifestyle (*OPAL*) community intervention program

**DOI:** 10.1186/s12889-019-7710-4

**Published:** 2019-10-22

**Authors:** Lucinda Bell, Shahid Ullah, Eva Leslie, Anthea Magarey, Timothy Olds, Julie Ratcliffe, Gang Chen, Michelle Miller, Michelle Jones, Lynne Cobiac

**Affiliations:** 1 Nutrition and Dietetics, College of Nursing and Health Sciences, Flinders University, GPO Box 2100, Adelaide, SA 5001 Australia; 2 Flinders Centre for Epidemiology and Biostatistics, School of Medicine, Flinders University, Bedford Park, South Australia Australia; 3 Australia and New Zealand Dialysis and Transplant (ANZDATA) Registry, South Australian Health and Medical Research Institute (SAHMRI), Adelaide Medical School, Faculty of Health and Medical Sciences, The University of Adelaide, Adelaide, South Australia Australia; 4 Alliance for Research in Exercise Nutrition and Activity (ARENA), University of South Australia, Adelaide, South Australia Australia; 5 Health Economics Unit, Repatriation General Hospital, Flinders University, Daws Park, South Australia Australia; 6 Institute for Choice, UniSA Business School, Adelaide, South Australia Australia; 7 College of Medicine and Public Health, Flinders University, Bedford Park, South Australia Australia; 8 OPAL (Obesity Prevention and Lifestyle), Public Health and Clinical Systems, SA Health, Adelaide, South Australia Australia; 9 Social Work, College of Education, Psychology and Social Work, Flinders University, Bedford Park, South Australia Australia; 10 CSIRO Health and Biosecurity, Adelaide, South Australia Australia

**Keywords:** Child, Obesity, Eating, Activity, Sedentary, Community, Intervention, Australia

## Abstract

**Background:**

Childhood obesity is a serious public health concern worldwide**.** Community-based obesity prevention interventions offer promise due to their focus on the broader social, cultural and environmental contexts rather than individual behaviour change and their potential for sustainability and scalability. This paper aims to determine the effectiveness of a South Australian community-based, multi-setting, multi-strategy intervention, *OPAL* (Obesity Prevention and Lifestyle), in increasing healthy weight prevalence in 9 to 11-year-olds.

**Methods:**

A quasi-experimental repeated cross-sectional design was employed. This paper reports on the anthropometric, health-related quality of life (HRQoL) and behaviour outcomes of primary school children (9–11 years) after 2–3 years of intervention delivery. Consenting children from primary schools (20 intervention communities, INT; 20 matched comparison communities, COMP) completed self-report questionnaires on diet, activity and screen time behaviours. HRQoL was measured using the Child Health Utility 9D. Body Mass Index (BMI) z-score and weight status were determined from children’s measured height and weight. A multilevel mixed-effects model, accounting for clustering in schools, was implemented to determine intervention effect. Sequential Bonferroni adjustment was used to allow for multiple comparisons of the secondary outcomes.

**Results:**

At baseline and final, respectively, 2611 and 1873 children completed questionnaires and 2353 and 1760 had anthropometric measures taken. The prevalence of children with healthy weight did not significantly change over time in INT (OR 1.11, 95%CI 0.92–1.35, *p* = 0.27) or COMP (OR 0.85, 95%CI 0.68–1.06, *p* = 0.14). Although changes in the likelihood of obesity, BMI z-score and HRQoL favoured the INT group, the differences were not significant after Bonferroni adjustment. There were also no significant differences between groups at final for behavioural outcomes.

**Conclusions:**

*OPAL* did not have a significant impact on the proportion of 9 to 11-year-olds in the healthy weight range, nor children’s BMI z-score, HRQoL and behaviours. Long-term, flexible community-based program evaluation approaches are required .

**Trial registration:**

ACTRN12616000477426 (12th April 2016, retrospectively registered).

## Background

Childhood obesity is a serious public health concern worldwide [1]. In Australia, at least 1 in 5 Australian children aged 2–16 years had overweight (17%) or obesity (6%) in 2007 [2]. In 2011–2012, these figures had risen to 1 in 4 children with overweight (19%) or obesity (7%) [3], highlighting the need for effective prevention strategies to stem, and reverse, the growth in childhood obesity in Australia. Specifically, with evidence of a greater burden of obesity in the most disadvantaged in developed countries [4], obesity-prevention strategies targeting socio-economically disadvantaged communities are required.

Despite childhood obesity prevention programs to date having shown, in general, moderate effectiveness in reducing adiposity [5], innovative, population-level approaches that are more effective, flexible, economical and, importantly, sustainable are urgently needed [6, 7]. Comprehensive community-based obesity prevention interventions offer promise due to their focus on the broader social, cultural and environmental contexts rather than individual behaviour change [8–11] and their potential for sustainability and scalability [7, 9, 12, 13]. Over the last decade community-based initiatives for childhood obesity prevention have begun to emerge, for example, the *Identification and prevention of Dietary- and lifestyle-induced health Effects In Children and InfantS (IDEFICS)* study in Europe [14], *Be Active Eat Well (BAEW)* [6, 7], *Eat Well Be Active (EWBA)* [15]*, Romp & Chomp* [8] and *It’s Your Move* [14] in Australia [6–8, 15, 16] and A Pilot Programme for Lifestyle and Exercise (APPLE) in New Zealand [17]. Such programs have shown positive impacts on anthropometric outcomes in pre-schoolers [15], primary school children [6, 17] and adolescents [8]. Modest improvements in diet and activity behaviours have also been observed [7, 15, 17–19]. Together, these findings demonstrate that community-based interventions can be effective [6].

The *EWBA Community Programs (2006–2009)* funded by, and based in, health services [16, 20] and engaging primarily in pre-schools and schools led the way for community-based obesity prevention in South Australia. Near the completion of *EWBA Community Programs* in 2008–2009, the South Australian government committed to the *OPAL* (Obesity Prevention and Lifestyle) program for 8 years, commencing in 2009/2010. *OPAL* was a systems-wide, multi-strategy, community-based childhood obesity prevention program funded by local, State and Australian governments and based in local government. It aimed to increase the proportion of 0–18 year olds in the healthy weight range, through families and *OPAL* communities, by creating supportive environments to improve eating and physical activity patterns of children. The *OPAL* program selected disadvantaged communities and was informed by social marketing, community development and social ecological systems theory. The latter is a framework that relates to the multiple environments within which an individual interacts, operationalised in *OPAL* as individuals, families, organisations, communities and environments [21, 22]. *OPAL* (in line with *BAEW* [6] and *EWBA* [16, 19]) was also underpinned by a community capacity building (CCB) approach, rather than applying a pre-developed program to the community. A CCB approach focuses on community participation, developing community ownership and enhancing skills in the ‘community’, to allow for sustainability, flexibility and scalability and to build the policies, environments and communities’ ethos over time [6]. Evidence on the effectiveness of community-based, capacity-building approaches such as *OPAL* are needed [7].

To determine the effectiveness of the *OPAL* approach, a comprehensive evaluation framework was developed [22, 23]. The primary *OPAL* evaluation outcome measures were changes in healthy weight prevalence and health-related quality of life (HRQoL). Secondary outcomes were eating and activity-related behaviours, attitudes and environments. The purpose of this paper is to report on the anthropometric (weight status and Body Mass Index (BMI) z-score), HRQoL and behaviour (diet, physical activity and sedentary behaviour) outcomes of primary school children aged 9–11 years from Phase 1 and 2 *OPAL* communities.

## Methods

### The OPAL intervention development and implementation

The *OPAL* program, which aimed to increase healthy weight prevalence in 0–18 year olds, was modeled on *EPODE* [24], (Ensemble, Prévenons l’Obésité des Enfants), a successful intervention from France which comprises political commitment, a scientific base, social marketing and partnerships [24–26]. *OPAL* targeted a wider age range than the French *EPODE* program [24] (children aged 0–12 years) and was implemented in both regional and metropolitan communities, whereas, at the time, *EPODE* had only been implemented in rural villages. *OPAL* was implemented in 20 lower socio-economic status (SES) communities (not necessarily geographically separate) in South Australia (Index of Relative Socio-economic Disadvantage (IRSD) [27], a measure of socio-economic status based on income- and education-related measures, was utilised and scores lower than 1000, i.e. average, preferenced). The program consisted of a central coordination unit with social marketing and evaluation expertise; together with local community teams consisting of two health promotion staff (Senior Manager and Support Officer) based in local council with a budget of AUD$75,000 per annum for 5 years (budget and staffing scaled to community population). With guidance from a Scientific Advisory Committee, the staff used a community development approach to develop and deliver annual social marketing theme messages and goal-related interventions to improve: 1) dietary intake and 2) physical activity. The intended mechanisms for reducing the consumption of energy-dense, nutrient-poor items and increasing healthy eating were to increase availability of healthy food in the home and at outlets and improving healthy food production, access and distribution [28]. The mechanisms for increasing physical activity and reducing sedentariness were to increase active travel, active leisure participation and use of parks and places [20].

The six social marketing themes were: 1) ‘Water. The original cool drink’ (February – August 2010), 2) ‘Give the screen a rest. Active play is best’ (September 2010 – April 2011), 3) ‘Make a fresh snack’ (May 2011 – January 2012), 4) ‘Think feet first. Step, cycle, scoot to school’ (February 2012 –January 2013), 5) ‘A healthy brekky is easy as Peel, Pour, Pop’ (February 2013 – February 2014) and 6) ‘Life looks brighter outside’ (March 2014 – June 2015) [29]. A suite of centrally coordinated social marketing materials was produced and complemented by Council-led activity with community stakeholders to create structural, program, educational and policy changes in support of the themes and goals [20]. As the *OPAL* program was shaped by each community’s needs, it was different in each community [20]. Examples of activities introduced in communities are; community gardens, installation of drinking water fountains in public places, extension of bike paths, improved facilities in sporting clubs, family fun days, and education and workplaces that promote healthy eating and physical activity programs [20].

### The OPAL evaluation design

**Fig. 1:**
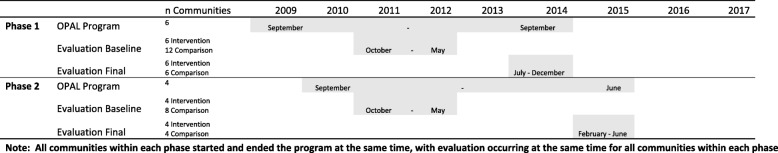
Dates of the *OPAL* program and *OPAL* Evaluation data collection for intervention (INT) and comparison (COMP) communities at baseline and final

A comprehensive evaluation framework (reported in detail elsewhere [21, 22, 26]) was developed to determine the effectiveness of the *OPAL* program. An internal evaluation manager oversaw the evaluation and this component of the evaluation was contracted to a local university for their high-level expertise and independence [21]. A quasi-experimental repeat cross-sectional design, involving cross-sectional surveys pre- and post-intervention (two samples) with intervention (INT) and non-randomized comparison communities (COMP), was used to obtain a series of ‘snapshots’ of the population at a particular point in time. Communities were the primary evaluation unit and a partial stepped wedge design was adopted. That is, in line with the staggered intake of communities into the *OPAL* program across four phases, the *OPAL* Evaluation was also staggered **(**Fig. 1**).**

Three age groups were initially selected for analysis from across the 0–18 year range: 1) 4–5 year olds (due to availability of child health check data); 2) 9 to 11-year-olds (pre-pubescent); and 3) 14–16 year olds (post-puberty). These age groups were selected to align with the planned 5-year evaluation period, with the intention of linking data (for example, 4–5 year old data with 9–11 year old) to create a longitudinal dataset. However, due to significant budget cuts to the OPAL program and evaluation on two occasions, limits were placed on the planned scale and scope of the final evaluation [21]. For example, in terms of scale, only the first 10 communities in Phases 1 (*n* = 6) and 2 (*n* = 4) were included at final **(**Table 1**).** Phase 1 *OPAL* intervention communities ran for a period of 5 years while Phase 2 ran for a shortened period of 4.75 years (Fig. 1). In terms of scope, a suite of surveys were collected at baseline from all three groups [25] while at final only routinely collected health-check data of 4–5 year olds was accessed, and child surveys, measures of 9 to 11-year-olds (grades 4–6) and parent surveys collected (no final evaluation of 14–16 year olds). Evaluation of 9–11 year-old children was preferenced to be retained as they are able to self-report and are less autonomous than older children and are therefore influenced by the community (for example, via community education and sporting programs), to whom the intervention was targeted. Further, due to initial delays in obtaining ethics permissions (delays in contractual agreements, negotiation of ethics approvals across multiple committees), baseline data were effectively collected ‘mid-term’ of the intervention (October 2011 – May 2012) and with the final evaluation concluding mid-2015 (July 2014–June 2015), the evaluation period was shortened to 2–3 years (Fig. 1**).**

**Table 1:** Surveys and measurements for students in Phase 1 and 2 intervention (INT) and comparison (COMP) communities

		Baseline		Final	
		n Survey	n Measures	n Survey	n Measures
Phase 1	Intervention	884	758	657	601
	Comparison	613	581	440	422
	Sub total	1497	1339	1097	1023
Phase 2	Intervention	489	450	435	409
	Comparison	625	564	341	328
	Sub total	1114	1014	776	737
Phase 1 & 2	Total	2611	2353	1873	1760

This paper reports the 9–11-year-old survey and measures for phase 1 and 2 communities at baseline and final **(**Table 1**)**. That is, a cross-sectional sample of 9 to 11-year-olds at baseline are compared to a sample of 9 to 11-year-olds at final who were exposed to the intervention 2–3 years prior. The study protocol was approved by the SA Health Human Research Ethics Committee, Flinders University Social and Behavioural Research Ethics Committee, the Department of Education and Children’s Services Research Unit, SA Catholic Education and Aboriginal Health Human Research Ethics Committee.

### The OPAL evaluation sample

The data collection methods from baseline are reported elsewhere [25]. At final, all primary (public and private) schools from 10 the South Australian OPAL communities (and 10 matched comparison communities) were invited to participate in the *OPAL* evaluation. An introductory letter was sent to primary school Principals from the Ministers for Health and Ageing, and Education and Child Development outlining the importance of the evaluation, seeking school-level consent and providing an information pack containing an information letter and brochure, checklist and participation form. A small monetary incentive ($50) was offered to participating schools at both baseline and follow up. Parents/guardians from consenting schools were invited to consent for their child, and themselves, to be involved in the evaluation. Students were also required to provide written and verbal assent to complete the survey and/or the measurements. Consenting children from INT and COMP provided data through self-report questionnaires (completed online or in hard copy) and anthropometric measures (completed on the same day as the questionnaires) obtained by trained data collectors (details provided below).

### Measures

#### Demographics

Socio-demographic data including age, sex, and postcode or town of residence were collected via child-completed questionnaire. School-level demographic data were also collected. Area of residence was classified as urban or rural, based on the location of the school the child attended and according to the Australian Bureau of Statistics (ABS) remoteness areas for Australia [30], where major cities of Australia were classified as urban; and inner and outer regional areas classified as rural. A measure of relative SES was determined using the Index of Community Socio-Educational Advantage (ICSEA) scores for schools [31], categorised as quintiles. ICSEA was created by the Australian Curriculum, Assessment and Reporting Authority (ACARA) from four characteristics: 1) socio-economic characteristics of the census collection districts where children in a school live, 2) whether a school is in a regional or remote area, 3) proportion of children from a language background other than English, and 4) the proportion of Aboriginal children enrolled at the school. ICSEA quintiles are based on the current national data in 2011 at baseline (cut-offs 940/980/1020/1076/1287) and 2014 at final (cut-offs 942/985/1023/1074/1292), where quintile 1 (Q1) represents schools at greatest socio-economic disadvantage and quintile 5 (Q5) represents schools at least socio-economic disadvantage.

#### Anthropometrics

Each consenting child was measured without shoes or heavy outer garments by trained data collectors, in line with the Body Image Guidelines developed and endorsed by the *OPAL* Scientific Advisory Committee. Data collectors were trained in body image, cultural sensitivities, mandatory reporting and anthropometry; one data collector on the team was required to be a registered teacher. Height (Invicta Stadiometer) and weight (Tanita BWB-800 portable electronic scales) measures were taken by the same data collector on one occasion and final measures determined as the mean of two measures, or the median if three measures were taken (in the case that the first two measures differed by more than 0.5 cm or 0.5 kg, for height and weight respectively). Body Mass Index (BMI) was calculated as weight (kg) divided by height (m) squared and converted to age- and sex-specific z-scores using the UK 1990 reference data [32]. Children were categorised as underweight (BMI z score < − 1 to <− 3), normal weight (0), overweight (> 1.0 - < 2.0) or obese (> 2.0) using the International Obesity Taskforce (IOTF) cut-points [33, 34].

#### Health-related quality of life

Health-related quality of life (HRQoL), a multidimensional construct that measures the impact of health or disease on physical and psychosocial functioning [35, 36], was measured using the Child Health Utility 9D (CHU9D) [37, 38]. The CHU9D is a generic preference-based HRQoL instrument designed specifically for application within cost utility analyses of health care treatment and preventive programs targeted at young people [37, 38]. The CHU9D was developed from its inception with young people and has been validated in children aged 7–11 years [38, 39] and 11–17 years [40–44]. The CHU9D contains a health state classification system which has nine dimensions: worried, sad, pain, tired, annoyed, schoolwork, sleep, daily routine, ability to join in activities, with five different levels representing increasing levels of severity within each dimension (i.e. responses from 1 to 5 where 1 indicates absence of any impairment and 5 indicates the most severe impairment). The CHU9D was scored using the newly developed Australian adolescent-specific scoring algorithm [45, 46]. The preference-based scoring algorithm (or called ‘tariff’, ‘value set’) emanating from the Australian adolescent population reflects the strength of preference on different health dimensions (e.g. physical versus mental psychosocial functioning) of the population. The overall HRQoL score derived from a preference-based instrument is called the health state utility and can be used to adjust the life years to calculate the quality adjusted life year (QALY). The utility scores are interpreted on the 0–1 (death-full health) QALY scale whereby lower utility scores indicate poorer HRQOL.

#### Diet

Within the OPAL questionnaire, children were asked to report the number of serves they consumed the previous day of vegetables (two questions), fruit (one question) and several discretionary foods and beverages (six questions/food groups), rather than ‘usual’ intake which children often have difficulty understanding. Photographs of serve sizes were provided to assist estimation. These questions were drawn, where possible, from existing instruments with either proven validity or reliability [13, 47] or which have been used in national [48] or state [2] surveys in order to provide comparability or benchmarking with *OPAL* evaluation findings. Vegetable intake referred to all potato, other vegetables and legumes and excluded fried potato (classified as a discretionary food [49]). Fruit intake excluded fruit juice. Children were classified according to whether they met the recommended intake (2 or more serves of fruit and 5 or more serves of vegetables) based on the revised food modelling of the Australian Dietary Guidelines [49].

A serve of each discretionary food was a standard portion or pre-packaged amount (e.g. can of sweetened beverage, muesli bar). The six food groups were: (i) sweetened beverages including soft drinks, cordial, (ii) fruit juice and fruit juice drinks, (iii) lollies, chocolate, fruit bars (iv) cakes, doughnuts, sweet biscuits, muffins, muesli bars (v) ice cream, icy poles, ice blocks, and (vi) savoury snacks and/or salty snacks (e.g. potato crisps, corn chips, barbecue-flavoured twists). Total intake of these foods was expressed as 600 kJ serve equivalents [49] based on the average portion size for the range of items included in each group, consistent with the Australian Guide to Healthy Eating [50]. For example, a serve of sweetened beverage was 125 ml orange juice or a 375 ml can of sweetened soft drink, which equate to 153 and 600 kJ respectively. As a proportion of 600 kJ the former represents 0.26 of a serve so a factor of 0.26 was applied to serves of fruit juice to add to serves of soft drink. As the new dietary guidelines modelling system [51] does not prescribe the number of serves of discretionary food that should be consumed according to age and sex the number recommended in the previous Australian Guide to Healthy Eating [50], namely two serves or fewer for 8–11 years, was used as a cut-point.

#### Physical activity and sedentary behaviour

To estimate the percentage of children meeting the physical activity recommendations (i.e. at least 60 min of moderate to vigorous physical activity each day) [52] children were asked “Over the last 7 days, on how many days were you physically active for a total of 60 min per day?” This question, and that for sedentary behaviour, were based on a validated item from the *Health Behavior of School Children Study* [53]*.* Sedentary behaviour was operationalised as screen time, reported to be an acceptable surrogate for overall level of sitting in children [54]. To estimate the percentage of children meeting the sedentary behaviour guideline (i.e. no more than 120 min of screen time (television, computer and videogame use) for entertainment each day [52]), children were asked “Over the last 7 days, on how many days did you get at least 120 minutes (or 2 hours) of screen time (TV, videogames or computer use) per day outside of school hours?”

### Statistical analysis

All statistical analyses were conducted using IBM SPSS Statistics version 22 (SPSS Inc., Chicago, IL, USA), STATA statistical software, version 14.0 [55], and R version 3.1.2 [56]. Means and standard deviations (SD’s) were calculated for continuous data and proportions for categorical data. The normality assumption was visually checked by frequency histogram and normal Q-Q plot for continuous measurements. The Anderson-Darling test was also performed to test the normality assumption. Given the study sample was not the full sample intended due to no final evaluation of Phase 3 and 4 children, a retrospective power calculation was undertaken. To address the 8% improvement of healthy weight children in the intervention group compared to the comparison group at final a total sample size of 2084 (1042 per group) was projected to provide power of 80% with an alpha level of 0.05, allowing for a 20% attrition rate.

Demographic data were analysed using t-tests or chi-squared tests. A multivariate multilevel mixed-effects model (two-level random slope model) was used to analyse outcomes due to the hierarchical structure of the data (children nested in schools). Thus, models were accounted for the clustering in schools using *xtmixed* (for interval scale data - BMI and BMI z-score) and *xtmelogit* (for binary outcomes – weight status). Schools were treated as random effects, and main effects were group (intervention or comparison), time (baseline and final) and group x time interaction. Weight status categories were treated as a series of dichotomous outcomes for example healthy weight vs. non-healthy weight (underweight, overweight, and obese), overweight vs. non-overweight (healthy weight, underweight, obese). Models were adjusted by age (as a child level characteristic; continuous variable) and ICSEA score (as a school level characteristic), as they were statistically significant in the univariate models and clinically important. Locality was not included due to the high multicollinearity with ICSEA score. Unadjusted estimates are also presented. In this paper, the proportion of healthy weight children was considered the primary outcome variable. Exploratory analyses of secondary outcomes (i.e. likelihood of obesity, BMI z-score, HRQoL, behaviours) were undertaken, with sequential Bonferroni adjustment used to allow for multiple comparisons. The level of significance was set at *p* < 0.05. Where appropriate, 95% confidence intervals (95% CIs) were reported along with *p*-values.

## Results

### Recruitment sample

Characteristics of the sample by community (INT and COMP) and according to a number of demographic factors are shown in Table 2. A total of 117 (per community, 1–20) and 94 (per community, 0–14) schools were recruited at baseline and final respectively. However, not all schools were visited due to, for example, staffing change and/or change in mind. Thus, a total of 2611 children from 111 schools (*n* = 6 communities phase 1, *n* = 4 communities phase 2) completed surveys at baseline (23% response rate; 56% response rate for schools) and 1873 children from 86 schools (*n* = 6 communities phase 1, *n* = 4 communities phase 2) completed surveys at final (21% response rate; 57% response rate for schools). Measures were taken on 2353 children at baseline (*n* = 3 no weight data, *n* = 4 no birthdate data) and 1760 at final (including 13 cases with height measures of ≤110 cm, deemed unrealistic [12/13 children were from the one school] and thus excluded from analyses). At both time points the average (SD) age of children was 10.6 (0.9) years, approximately half female (50.2% baseline, 52.8% final) and there were higher proportions recruited from urban locations (66% baseline, 69% final) than from rural locations (34% baseline, 31% final). Significant differences were observed between INT and COMP at baseline and final in SES (Q1 – Q5; *p* < 0.001, higher SES in COMP compared with INT at both time points) and locality (urban, rural; *p* < 0.001, more urban children in INT at baseline and in COMP at final). Overall, more than one fifth of students at baseline (21.7%) and nearly a quarter at final (23.9%) were overweight or obese. Nearly three-quarters were of healthy weight (baseline 71.7%, final 70.0%).

**Table 2:** Characteristics of the children who completed questionnaires

	Year 3 (Baseline) (*n* = 2611)		Statistical difference^a^	Year 5 (Final) (*n* = 1873)		Statistical difference^b^
	INT (n, %)	COMP (n, %)	*P* value	INT (n, %)	COMP (n, %)	*P* value
All	1373 (52.6)	1238 (47.4)		1092 (58.3)	781 (41.7)	
Sex			0.199			0.040
Boys	700 (51.0)	600 (48.5)		490 (44.9)	388 (49.7)	
Girls	673 (49.0)	638 (51.5)		602 (55.1)	393 (50.3)	
Locality^c^			< 0.001			< 0.001
Urban	965 (70.3)	741 (59.9)		705 (64.6)	574 (73.7)	
Rural	408 (29.7)	497 (40.1)		387 (35.4)	205 (26.3)	
Age, years			0.051			0.125
≤9	374 (27.3)	379 (30.6)		340 (31.1)	214 (27.4)	
10	481 (35.1)	447 (36.1)		380 (34.8)	270 (34.6)	
≥11	514 (37.5)	412 (33.3)		372 (34.0)	297 (38.0)	
SES^d^			< 0.001			< 0.001
Quintile 1	271 (19.7)	88 (7.1)		268 (24.5)	54 (6.9)	
Quintile 2	421 (30.7)	220 (17.8)		217 (19.9)	140 (17.9)	
Quintile 3	328 (23.9)	198 (16.0)		251 (23.0)	223 (28.6)	
Quintile 4	237 (17.3)	607 (49.0)		334 (30.6)	364 (46.6)	
Quintile 5	116 (8.4)	124 (10.0)		22 (2.0)	0 (0)	
Phase			< 0.001			0.097
1	884 (64.4)	613 (49.5)		657 (60.2)	440 (56.3)	
2	489 (35.6)	625 (50.5)		435 (39.8)	341 (43.7)	

*Abbreviations*: *INT* Intervention communities, *COMP* Comparison communities

^a^Difference between INT and COMP at baseline; ^b^Difference between INT and COMP at final; ^c^*n* = 1 missing at final in COMP; ^d^SES is measured by ICSEA scores. Quintiles (Q1 = highest, Q5 = lowest) are based on 2011 National data at baseline (cut-offs 940/980/1020/1076/1287) and 2014 National data at final (cut-offs 942/985/1023/1074/1292). The national average ICSEA score is 1000 [31]. NB: As ICSEA score is not an individual-level SES measure but a school-level measure [31], caution should be taken when interpreting these data. Importantly, ICSEA does not use individual information concerning the wealth of the parents or children

### Changes in anthropometric measures

Table 3 shows the anthropometric details of the sample at each time point. When adjusted by age and ICSEA score, the proportion of children in the healthy weight range did not significantly change over the 2–3-year *OPAL* intervention period; (difference at final, OR 1.31, 95%CI 0.98–1.76, *p* = 0.07). Exploratory analyses of secondary outcomes revealed there were no significant differences in the proportion of children with overweight (baseline v final; 18% v 19% INT, 16 v 18% COMP) or combined overweight/obesity (baseline v final; 24% v 23% INT, 20 v 24% COMP) or between the INT and COMP groups over the intervention period. At final there was a 49% lower likelihood of children with obesity (not including overweight) from INT (baseline v final, 6% v 5%) than from COMP (baseline v final, 4% v 7%) (OR = 0.51, 95%CI 0.28–0.92, *p* = 0.03). However, this was no longer significant after adjustment for multiple comparisons. The difference in change in BMI z-score over-time between INT and COMP was not statistically significant (− 0.08, 95%CI -0.24 – 0.08, *p* = 0.31).

**Table 3:** Anthropometry, HRQOL and proportion (%) of children in each weight status category^a^ for total sample

	INT		COMP		Intervention effect (unadjusted models) (marginal mean difference ^b^)				Intervention effect (adjusted models)^c^ (marginal mean difference^b^)			
	Y3 (Baseline)	Y5 (Final)	Y3 (Baseline)	Y5 (Final)	INT	COMP	INT vs COMP	*p* value	INT	COMP	INT vs COMP	*p* value
*n*	1208	1010	1145	750	1208	1010	1145	750				
Anthropometry												
BMI (kg/m^2^)	18.54^d^	18.43^e^	18.29^e^	18.64^e^	−0.17 (−0.47–0.13)	0.37 (−0.01–0.76)	−0.54 (−1.03 - -0.05)	0.029**	−0.12 (−0.40–0.17)	0.16 (−0.17–0.50)	−0.28 (−0.72–0.16)	0.209
BMI z-score	0.33^g^	0.40^f^	0.30^e^	0.46^e^	0.05 (− 0.05–0.16)	0.18** (0.04–0.31)	− 0.12 (− 0.29–0.05)	0.151	0.05 (− 0.05–0.16)	0.14** (− 0.02–0.26)	−0.08 (− 0.24–0.08)	0.306
HRQoL	0.80	0.79	0.82	0.77	–	–	–	–	−0.012 (− 0.029–0.004)	−0.046** (− 0.071 – − 0.022)	0.034** (0.006–0.062)	0.016
	INT		COMP		Intervention effect (unadjusted models) (odds ratio (95% CI)^2^)				Intervention effect (adjusted models) (odds ratio (95% CI)^2^)			
	Y3 (Baseline)	Y5 (Final)	Y3 (Baseline)	Y5 (Final)	INT^h^	COMP^i^	INT vs COMP^j^	*p* value	INT^h^	COMP^i^	INT vs COMP^j^	*p* value
*n*	1202	983	1144	749	1202	983	1144	749				
Weight status category^a^	%	%	%	%								
Underweight	7.4	4.7	5.8	4.4	0.62** (0.43–0.91)	0.75 (0.48–1.18)	0.83 (0.46–1.48)	0.525	0.63** (0.43–0.91)	0.75 (0.48–1.17)	0.84 (0.47–1.52)	0.570
Healthy weight	69.1	70.9	74.5	70.5	1.13 (0.92–1.37)	0.78 (0.61–1.00)	1.44 (1.05–1.98)	0.024**	1.11 (0.92–1.35)	0.85 (0.68–1.06)	1.31 (0.98–1.73)	0.069
Overweight	18.1	18.7	15.8	17.9	0.99 (0.78–1.26)	1.25 (0.94–1.68)	0.79 (0.54–1.16)	0.226	1.01 (0.80–1.27)	1.16 (0.88–1.54)	0.87 (0.60–1.25)	0.451
Obese	5.5	4.6	3.9	6.5	0.85 (0.56–1.27)	1.65** (1.03–2.64)	0.51** (0.28–0.95)	0.035**	0.81 (0.54–1.21)	1.61** (1.03–2.51)	0.51** (0.28–0.92)	0.026**
Combined overweight/obese	23.5	23.3	19.8	24.4	0.94 (0.75–1.17)	1.41** (1.07–1.86)	0.67 (0.47–0.95)	0.025**	0.96 (0.78–1.18)	1.27 (1.00–1.62)	0.75 (0.54–1.04)	0.083

*Abbreviations INT* Intervention communities, *COMP* Comparison communities, *Y* Year

**p < 0.05 before Bonferroni adjustment. No comparisons were significantly different after sequential Bonferroni adjustment

^a^International Obesity Taskforce cut-points [33, 34]; ^b^Marginal mean difference for continuous measures and odds ratio form weight status categories; ^c^Models were adjusted by age and ICSEA score, other than BMI z-score which was adjusted for ICSEA score only; ^d^*n* = 2 missing; ^e^*n* = 1 missing; ^f^*n* = 12 missing; ^g^*n* = 6 missing; ^h^Odds of weight status categories at final for intervention group, baseline is the reference group; ^i^Odds of weight status categories at final for comparison group, baseline is the reference group; ^j^Odds of weight status categories for INT^8^, COMP^9^ is the reference group

### Change in HRQoL

A decreasing trend on CHU9D utilities was observed for both INT (0.804 baseline, 0.792 final; adjusted difference = − 0.012, *p* = 0.139) and COMP (0.820 baseline, 0.766 final; adjusted difference = − 0.054, *p* < 0.001). On average, at the final time-point children from INT had an adjusted (by age and ICSEA score) incremental mean utility gain of ∆ = 0.034 (95%CI 0.006–0.062, *p* = 0.02) when compared to children from COMP. This was no longer significant after adjustment for multiple comparisons.

### Change in behaviours

Exploratory analyses of secondary behavioural outcomes revealed that the probability of children meeting the recommended fruit (OR = 1.3 95%CI 0.9–1.7, *p* = 0.08) and vegetable (OR = 0.8 95%CI 0.6–1.1, *p* = 0.19) intake was not statistically different between INT (baseline v final; 58% v 66% fruit, 18% v 21% vegetables) and COMP (baseline v final; 67% v 71% fruit, 17% v 24% vegetables) at final **(**Table 4**).** The probability of children meeting the discretionary food guideline increased by 40% in INT (baseline v final, 24% v 28%) compared to children from COMP (baseline v final, 29% v 25%) (OR = 1.4, 95%CI 1.0–1.9, *p* = 0.04). However, this was no longer significant after adjustment for multiple comparisons. Children were 60% more likely to meet the physical activity guidelines at final than baseline in both INT (baseline v final, 28% v 37%) and COMP (baseline v final, 28% v 40%); the difference between groups at final was not statistically significant (OR = 1.0, 95%CI 0.7–1.3, *p* = 0.97). Children from both groups were less likely to meet the screen time guidelines at final (INT 13%, COMP 11%) than baseline (INT 17%, COMP 20%), with the difference between groups at final not statistically significant (OR = 1.4, 95%CI 0.9–2.0, *p* = 0.05).

**Table 4:** Proportion (%) of children meeting recommendations

	INT		COMP		OR (95%CI) (Year 3 – Year 5)		
	Y3 (Baseline)	Y5 (Final)	Y3 (baseline)	Y5 (final	INT^a^	COMP^b^	INT vs COMP^c^
Fruit (2 serves)^d^							
n	1356	1090	1231	776			
%	57.7	66.3	67.4	70.8	1.5* (1.3–1.8)	1.2 (0.9–1.5)	1.3 (0.9–1.7)
Vegetables (5 serves)^e^							
n	1327	1090	1188	774			
**%**	17.6	20.6	16.8	23.5	1.2 (1.0–1.5)	1.5* (1.2–1.9)	0.8 (0.6–1.1)
Discretionary food (2 serves or less)							
n	1320	1090	1206	774			
**%**	24.1	27.8	29.2	24.8	1.2 (1.0–1.4)	0.9 (0.7–1.1)	1.4** (1.0–1.9)
Physical activity (≥60 mins/d)							
n	1359	1092	1227	777			
**%**	27.7	37.0	28.3	39.9	1.6* (1.3–1.9)	1.6* (1.2–2.0)	1.0 (0.7–1.3)
Screen time (< 120 min/d)							
n	1346	1090	1210	777			
**%**	17.1	12.8	19.8	10.9	0.7* (0.6–0.9)	0.5* (0.4–0.7)	1.4 (0.9–2.0)

*Abbreviations*: *INT* Intervention communities, *COMP* Comparison communities

*p < 0.01; ** *p* < 0.05

^a^Odds at final for intervention group (INT), baseline is the reference; ^b^Odds at final for comparison group (COMP), baseline is the reference group; ^c^Odds for the intervention group (INT), the comparison group (COMP) is the reference group. Note: Models were adjusted by age and ICSEA score

^d^Fruit estimates exclude fruit juice; ^e^Vegetable estimates include potatoes (excluding fried potatoes)

## Discussion

This paper reports the findings of the evaluation of the *OPAL* (Obesity Prevention and Lifestyle) program, a systems-wide, multi-strategy, community-based program based in local government which aimed to increase prevalence of healthy weight and HRQoL among children aged 0–18 years by improving diet and activity behaviours. Overall, there was no significant intervention effect on the proportion with healthy weight or BMI z-score amongst primary school children aged 9–11 years. The reduced probability of children with obesity, increased probability of children meeting the discretionary food guideline (i.e. improved intake), and improved HRQoL at the end of the intervention period amongst intervention children were no longer significant after adjustment for multiple comparisons.

### Intervention impact on children’s anthropometrics

Despite exploratory analysis revealing a 49% lower probability of children with obesity in intervention compared to comparison communities at the end of the 2–3 year evaluation period (not significant after adjustment for multiple comparisons), the *OPAL* program did not result in a significant difference in BMI z-score (− 0.08 points) or proportion with healthy weight in 9–11 year old children. These findings are similar to those reported previously for local community-based initiatives, yet different to interstate and international initiatives. That is, the South Australian *EWBA community programs* (2006–2009), which also utilised a quasi-experimental repeat cross-sectional design across 3 years), did not result in significant changes in BMI z-score or weight status in 10–12 year old children [16]. In comparison, the *BAEW* [6] (a quasi-experimental longitudinal study in Victoria, Australia) and *APPLE* [17] programs (a 2-year controlled community-based intervention in New Zealand) resulted in significant reductions in BMI z-score in children aged 4–12 years [6] (− 0.11, 95%CI -0.21, − 0.01) and 5–12 years [17] (at both 1 [− 0.09, 95%CI -0.18, − 0.01] and 2 year [− 0.26, 95%CI -0.32, − 0.21] follow-ups), respectively. The similar effects seen in *APPLE* [17], *BAEW* [6] and *OPAL* on BMI z-score (approximately 0.1 units over 3 years; not significant for *OPAL*) demonstrate the potential for effectiveness of community-based obesity prevention interventions. Yet, the consistent lack of intervention effect on prevalence of children with overweight or obesity in these studies ([6, 17] including OPAL) may reflect the fact that categorical weight status is a blunter measure of change than continuous measures such as BMI z-score [6] and/or the comparatively short intervention (and evaluation) periods (2–3 years). For example, the Fleurbaix-Laventie Ville Santé (FLVS) study in France [25], on which *EPODE* was based [24], saw a downward trend in children with overweight after 12 years of school and community programs [25]. Long-term evaluation is thus warranted.

### Intervention impact on children’s HRQoL

Given obesity, and the underlying behaviours (eating, activity and sedentary behaviour), can influence children’s psychosocial functioning [57], the intervention impact on children’s HRQoL was evaluated. Despite a relatively improved HRQoL amongst intervention children compared to comparison children. and a downward trend on CHU9D utilities in both groups, between-group comparisons were not significant after adjustment for multiple comparisons. Few studies have examined the impact of community-based obesity prevention programs on primary school children’s HRQoL. The *APPLE* program found no significant difference between intervention and control children’s Health Utility Index scores (parental proxy used to represent HRQoL) after the 2-year intervention, despite a significant intervention impact on BMI z-scores [58]. These differential results may be partly explained by the choice of HRQoL instrument. That is, APPLE utilised the Health Utilities Index, a generic preference-based measure of HRQoL originally developed for adults, whereas *OPAL* utilised the CHU9D, a measure developed with young people [59] and thus more likely to be sensitive to quality of life dimensions most pertinent for young people. Our findings indicate a more positive impact on HRQOL, with children from intervention communities gaining in health state utility when compared to comparison children, and thus demonstrate the added value of including this outcome measure (alongside anthropometric measures) to provide evidence on the effectiveness (including cost-effectiveness) of future child obesity prevention initiatives.

### Intervention impact on children’s lifestyle behaviours

Overall, few positive behaviour changes were observed in this study. There was no intervention effect on the proportion of children meeting the fruit or vegetable guidelines. This is similar to that observed in *EWBA* [19] but in contrast to the *BAEW* program [7] which resulted in significant improvements in fruit intake at home [7, 18] (but not on fruit and vegetables brought to school [18]), and the *APPLE* program which had a significant effect on fruit, but not vegetable intake [17]. The slightly better findings observed in *BAEW* [7] and *APPLE* [17] compared to *OPAL* may be because *OPAL*’s nutrition-related messages did not focus specifically on fruit and vegetables, but rather encouraged the replacement of discretionary food snacks with healthy options through the social marketing theme ‘make it a fresh snack’. This may further explain the intervention effect on children meeting the discretionary food guideline (i.e. improved food intake) in *OPAL* (incomparable measures used in other studies). However, given this was no longer significant after adjustment for multiple comparisons, findings should be interpreted with caution. Although improvements in children’s physical activity and screen time behaviours were observed in *OPAL* across both intervention and comparison communities, no significant difference was found between groups. This is similar to *BAEW* [18] and *EWBA* [19] which observed no significant differences between groups in TV watching [18], playing computer games [18], screen time [19] and physical activity [19]. However, intervention children spent more time playing outside after school after the *BAEW* intervention [18] (compared to less time in comparison children), and had significantly higher accelerometer counts than control children at 1 (but not 2) years of the APPLE program [17]. It is possible that these changes in physical activity in *BAEW* and *APPLE* contributed to the significant impacts on BMI z-score observed in these programs, although these studies were not designed, nor powered, to identify whether changes in these components explain changes in anthropometric measures [17].

The behaviour findings reported here should be interpreted with some caution given the crude nature of the assessment tools used across studies and differences in criteria applied for meeting/not meeting behaviour guideline. Importantly, the self-report measures used in OPAL also required a level of literacy and cognitive ability that was not achievable by some children, evidenced by the removal of implausible results, which may have affected the accuracy of reporting and thus the outcomes observed in this study. Nonetheless, the lack of a consistent effect on dietary (fruit, vegetables and discretionary intake) and behaviour (activity, screen time) across several community-based programs (*OPAL, BAEW, EWBA*), despite the inclusion of healthy eating and activity messages, highlights the need for more effective ways of changing consumption towards healthier eating patterns [18], in particular vegetable consumption, and screen time activities.

### The success of the OPAL program

In contrast to previous community-based initiatives, *OPAL* did not have a significant impact on the proportion of 9 to 11-year-olds in the healthy weight range, nor children’s BMI z-score, HRQoL, and behavioral outcomes. There are several possible reasons for these findings. Although *the CCB* approach has been shown to be effective when applied to one purposively selected rural community (Colac, Victoria, Australia; *BAEW* [6, 7]) or several small rural villages in France (population, *n* = 6500 people; *FLVS*), it was less effective when applied to multiple and mixed metropolitan and regional communities in *EWBA* [16, 19] (*n* = 2) *and OPAL* (*n* = 10). This may suggest that a CCB approach works better in rural and/or smaller communities, possibly due to better spread of messages via word-of-mouth in smaller communities, wider coverage of initiatives due to fewer diet and/or physical activity institutions such as supermarkets or sports clubs, and/or lower risk of contamination between communities. Yet, other variations between programs must also be considered, including: the level of support (part-funding, in-kind funding) from local councils; the ‘dose’ of the intervention; the level of investment in staffing and program activity; and population targets (where *OPAL* had the widest population target of 0–18 years and thus potentially the lowest program dose).

The broader sociocultural-political context within which *OPAL* operated may have also impacted on the success of the program. Although *OPAL* was the only program to incorporate social marketing campaigns, this occurred within an environment saturated with multi-national companies advertising energy-dense nutrient-poor foods directly to children [60]. There were also some healthy options campaigns that ran nationally (for example, for Australian bananas), and state-wide (Go for 2&5® fruit and vegetable campaign), which may have diluted the intervention effect due to being advertised in both intervention and comparison communities. Politically, in 2012 the McCann review of South Australian health promotion services [61] resulted in substantial cuts to the sector and in 2013, at the Federal level, the government change minimised the prevention and primary health care agendas [62, 63]. The effects of the sociocultural-political context on the *OPAL* program cannot be under-estimated.

### Strengths and limitations

Limitations of the evaluation may have also contributed to the lack of intervention effect seen in OPAL. For example, the evaluation sample size (~ 1900-2600) is modest compared to, for example, > 16,000 children in the IDEFICS study in Europe [14], although larger than in *EWBA* (~ 1000 – 1200) [16, 19] and comparable to *BAEW* (~ 2000), and thus there may not have been enough power to detect significant differences between groups. The relatively short (2–3 year) evaluation period, although similar to previous evaluations [6, 16], may not have been long enough to see significant changes in the outcomes measured, in particular weight-related outcomes. The term ‘baseline’ should also be treated with caution as baseline data were collected at year 2–3 (not year 0) of the intervention. The effect of these limitations on the outcomes observed cannot be underestimated.

In addition, the evaluation was also not able to measure the dose of the *OPAL* intervention received by children and parents within intervention communities, nor how well it was adopted [64]. Nonetheless, assessment of implementation fidelity and adaptation were assessed in phase 2 OPAL communities with nearly three quarters (70%) of all strategies implemented with integrity [64]. However, cross-contamination of *OPAL* messages between intervention and comparison may have occurred, for example in those children who lived in a comparison community but attended school in an intervention community, or vice versa. Further, the modest response rates (less than 25%), lower than those reported in other community-based child obesity prevention interventions in Australia (of approximately 50% [6, 7, 16]), possibly due to selection of *OPAL* communities according to higher levels of disadvantage, limits the power (and thus reliability) and representativeness of these findings. Nonetheless, the age and sex distribution of children at baseline and final, and the prevalence of children with overweight and obesity in the sample (23%), were similar to national (28%, 9–13 years) [48] and state (23%) [2] surveys, providing some confidence in the generalisability of the findings. Despite the intention of matching intervention and comparison communities according to SES, the inability to recruit sufficient low SES communities as ‘matched’ comparisons resulted in large disparities in SES between communities.

Importantly, measures may not have been robust enough to capture the impact of a multi-component community-based trial. Although questionnaire items assessing behaviours were mostly adapted from validated questionnaires [13, 47, 53], or national [48] or state [2] surveys to allow for comparability, the psychometric properties of *OPAL* questionnaires have not been tested due to budget and time restrictions. Further, due to the complexity of the evaluation and the pragmatism that needed to be applied, dietary data were based on 1 day of intake and therefore do not reflect ‘usual’ eating patterns. The anthropometric analysis is also limited by the use of dichotomous weight status outcomes in the multilevel mixed effect model which results in a loss of precision and power. Lastly, due to the cross-sectional nature of measurements taken in the same communities at two time points, rather than upon specific individuals, changes observed cannot be solely attributed to the OPAL intervention. Given these measurement limitations, conclusions should be drawn with caution.

Nonetheless, the strengths of this study include the comprehensive quasi-experimental evaluation, with matched comparison communities, of children’s weight status, HRQoL, and lifestyle behaviours using objective, self-report measures in a relatively large sample. Another major strength is the site-specific adaptation of a previously successful community-based childhood obesity prevention intervention (EPODE) and the use of a community capacity-building approach rather than applying a pre-developed intervention program to a community.

### Lessons learnt and future directions

This study demonstrates the complexity of evaluating complex, multi-component, community-based interventions under real-world conditions. The quasi-experimental evaluation design used in this study is associated with more risk of bias than individual or setting-based randomization. Although the inclusion of comparison communities helps to reduce this bias, communities were not blinded to group allocation. Alternative research designs such as ecological and longitudinal cohort models, should be considered in future evaluations given fidelity to an intervention in ‘real-life conditions’ is difficult, as is identifying the ‘active’ ingredient in a mix of interventions. Thus more adaptive/flexible evaluation approaches may be required in future to measure cross-contamination. Future evaluations should also consider: (1) opt-out, rather than opt-in, consent to increase response rates and thus improve statistical power; (2) ways to improve recruitment through schools, such as investing time in establishing strong relationships, minimizing burden, and providing substantial rewards for participation; and (3) low-touch, high-tech measurement methods, such as accelerometry, wherever possible to measure 24-h activity compositions. Of importance is that despite attempts in *OPAL* and *EWBA* to intervene with parents (the gatekeepers of children’s food and activity environment in early childhood and early primary school [65]) via the child, direct intervention was not possible. Intervening directly with the parents, in addition to the broader community, remains an untapped area.

Further, given a 3-year evaluation period was observed in OPAL (despite a planned 5-year evaluation), the sustainability or long-term changes have not been assessed in these communities and therefore is unknown. It is possible that the improvement in HRQoL seen in this study, in addition to the intervention effect on children meeting the discretionary food guideline in *OPAL* (incomparable measures used in other studies), may lead to an intervention effect on BMI z score and/or overweight and obesity prevalence, in the long term. Evidently, the impact of the short evaluation period on the findings reported here, cannot be underestimated. Thus, future programs should invest in ensuring community leaders and politicians understand the requirements of scientific trials, particularly with regards to project duration, as well as randomisation, consistency of treatment/fidelity, and avoiding contamination. The effectiveness of future programs may also depend on continued tripartite government support (Local, State and Federal), supported by a whole-of-government approach across all departments (e.g. Education and Health), as well as from councils (a key feature of the *OPAL* program) in establishing protocols and recruitment processes. The establishment of routine data collections for anthropometric measures across childhood, as is being conducted in the United Kingdom (National Child Measurement Program [66]), and more streamlined ethics processes, would assist in overcoming the challenges experienced in evaluating the OPAL program.

## Conclusions

These findings contribute to the understanding of the effectiveness of community-based childhood obesity prevention programs. The *OPAL* program utilised a range of strategies implemented across many settings. Although the proportion of healthy weight children did not significantly change over the 2–3 year evaluation period, the maintenance of the prevalence of children with overweight/obesity in intervention communities is encouraging. Further, although changes in the likelihood of obesity, BMI z-score and HRQoL were not significant after Bonferroni adjustment, changes favoured the intervention group, with a reduction in the probability of children with obesity observed beyond what other similar complex community-level interventions have previously achieved and a promising small non-significant improvement in BMI z-score in intervention communities. Yet the impact of the shorter-than-envisaged evaluation period, and the lack of a true baseline, on the findings cannot be underestimated. Importantly, this study adds to our understanding of the challenges associated with implementing and evaluating complex community-based initiatives. More flexible community-level evaluation approaches that overcome issues with recruitment, measurement and cross-contamination, are required.

## Data Availability

The data that support the findings of this study are available from the South Australian Department of Health. However, restrictions apply to the availability of these data, which were under license for the current study, and so are not publicly available.

## Abbreviations

APPLE: A Pilot Program for Lifestyle and Exercise
BAEW: Be Active Eat Well
EWBA: Eat Well Be Active
OPAL: Obesity Prevention and Lifestyle
